# Differences in space weathering between the near and far side of the Moon: evidence from Chang'e-6 samples

**DOI:** 10.1093/nsr/nwaf087

**Published:** 2025-03-05

**Authors:** Jiarui Lin, Haiyang Xian, Yiping Yang, Shan Li, Jiaxin Xi, Xiaoju Lin, Yao Xiao, Shengdong Chen, Chenyi Zhao, Miaomiao Zhang, Akira Tsuchiyama, Jianxi Zhu, Hongping He, Yi-Gang Xu

**Affiliations:** State Key Laboratory of Deep Earth Processes and Resources, Guangzhou Institute of Geochemistry, Chinese Academy of Sciences, Guangzhou 510640, China; Center for Advanced Planetary Science (CAPS), Guangzhou Institute of Geochemistry, Chinese Academy of Sciences, Guangzhou 510640, China; Guangdong Provincial Key Laboratory of Mineral Physics and Materials, Guangzhou Institute of Geochemistry, Chinese Academy of Sciences, Guangzhou 510640, China; University of Chinese Academy of Sciences, Beijing 101408, China; State Key Laboratory of Deep Earth Processes and Resources, Guangzhou Institute of Geochemistry, Chinese Academy of Sciences, Guangzhou 510640, China; Center for Advanced Planetary Science (CAPS), Guangzhou Institute of Geochemistry, Chinese Academy of Sciences, Guangzhou 510640, China; Guangdong Provincial Key Laboratory of Mineral Physics and Materials, Guangzhou Institute of Geochemistry, Chinese Academy of Sciences, Guangzhou 510640, China; State Key Laboratory of Deep Earth Processes and Resources, Guangzhou Institute of Geochemistry, Chinese Academy of Sciences, Guangzhou 510640, China; Center for Advanced Planetary Science (CAPS), Guangzhou Institute of Geochemistry, Chinese Academy of Sciences, Guangzhou 510640, China; Guangdong Provincial Key Laboratory of Mineral Physics and Materials, Guangzhou Institute of Geochemistry, Chinese Academy of Sciences, Guangzhou 510640, China; State Key Laboratory of Deep Earth Processes and Resources, Guangzhou Institute of Geochemistry, Chinese Academy of Sciences, Guangzhou 510640, China; Center for Advanced Planetary Science (CAPS), Guangzhou Institute of Geochemistry, Chinese Academy of Sciences, Guangzhou 510640, China; Guangdong Provincial Key Laboratory of Mineral Physics and Materials, Guangzhou Institute of Geochemistry, Chinese Academy of Sciences, Guangzhou 510640, China; University of Chinese Academy of Sciences, Beijing 101408, China; State Key Laboratory of Deep Earth Processes and Resources, Guangzhou Institute of Geochemistry, Chinese Academy of Sciences, Guangzhou 510640, China; Center for Advanced Planetary Science (CAPS), Guangzhou Institute of Geochemistry, Chinese Academy of Sciences, Guangzhou 510640, China; Guangdong Provincial Key Laboratory of Mineral Physics and Materials, Guangzhou Institute of Geochemistry, Chinese Academy of Sciences, Guangzhou 510640, China; University of Chinese Academy of Sciences, Beijing 101408, China; State Key Laboratory of Deep Earth Processes and Resources, Guangzhou Institute of Geochemistry, Chinese Academy of Sciences, Guangzhou 510640, China; Center for Advanced Planetary Science (CAPS), Guangzhou Institute of Geochemistry, Chinese Academy of Sciences, Guangzhou 510640, China; Guangdong Provincial Key Laboratory of Mineral Physics and Materials, Guangzhou Institute of Geochemistry, Chinese Academy of Sciences, Guangzhou 510640, China; State Key Laboratory of Deep Earth Processes and Resources, Guangzhou Institute of Geochemistry, Chinese Academy of Sciences, Guangzhou 510640, China; Center for Advanced Planetary Science (CAPS), Guangzhou Institute of Geochemistry, Chinese Academy of Sciences, Guangzhou 510640, China; Guangdong Provincial Key Laboratory of Mineral Physics and Materials, Guangzhou Institute of Geochemistry, Chinese Academy of Sciences, Guangzhou 510640, China; University of Chinese Academy of Sciences, Beijing 101408, China; State Key Laboratory of Deep Earth Processes and Resources, Guangzhou Institute of Geochemistry, Chinese Academy of Sciences, Guangzhou 510640, China; Center for Advanced Planetary Science (CAPS), Guangzhou Institute of Geochemistry, Chinese Academy of Sciences, Guangzhou 510640, China; Guangdong Provincial Key Laboratory of Mineral Physics and Materials, Guangzhou Institute of Geochemistry, Chinese Academy of Sciences, Guangzhou 510640, China; University of Chinese Academy of Sciences, Beijing 101408, China; State Key Laboratory of Deep Earth Processes and Resources, Guangzhou Institute of Geochemistry, Chinese Academy of Sciences, Guangzhou 510640, China; Center for Advanced Planetary Science (CAPS), Guangzhou Institute of Geochemistry, Chinese Academy of Sciences, Guangzhou 510640, China; Guangdong Provincial Key Laboratory of Mineral Physics and Materials, Guangzhou Institute of Geochemistry, Chinese Academy of Sciences, Guangzhou 510640, China; State Key Laboratory of Deep Earth Processes and Resources, Guangzhou Institute of Geochemistry, Chinese Academy of Sciences, Guangzhou 510640, China; Center for Advanced Planetary Science (CAPS), Guangzhou Institute of Geochemistry, Chinese Academy of Sciences, Guangzhou 510640, China; Guangdong Provincial Key Laboratory of Mineral Physics and Materials, Guangzhou Institute of Geochemistry, Chinese Academy of Sciences, Guangzhou 510640, China; State Key Laboratory of Deep Earth Processes and Resources, Guangzhou Institute of Geochemistry, Chinese Academy of Sciences, Guangzhou 510640, China; Center for Advanced Planetary Science (CAPS), Guangzhou Institute of Geochemistry, Chinese Academy of Sciences, Guangzhou 510640, China; State Key Laboratory of Deep Earth Processes and Resources, Guangzhou Institute of Geochemistry, Chinese Academy of Sciences, Guangzhou 510640, China; Center for Advanced Planetary Science (CAPS), Guangzhou Institute of Geochemistry, Chinese Academy of Sciences, Guangzhou 510640, China; Guangdong Provincial Key Laboratory of Mineral Physics and Materials, Guangzhou Institute of Geochemistry, Chinese Academy of Sciences, Guangzhou 510640, China; State Key Laboratory of Deep Earth Processes and Resources, Guangzhou Institute of Geochemistry, Chinese Academy of Sciences, Guangzhou 510640, China; Center for Advanced Planetary Science (CAPS), Guangzhou Institute of Geochemistry, Chinese Academy of Sciences, Guangzhou 510640, China; Guangdong Provincial Key Laboratory of Mineral Physics and Materials, Guangzhou Institute of Geochemistry, Chinese Academy of Sciences, Guangzhou 510640, China; University of Chinese Academy of Sciences, Beijing 101408, China; State Key Laboratory of Deep Earth Processes and Resources, Guangzhou Institute of Geochemistry, Chinese Academy of Sciences, Guangzhou 510640, China; Center for Advanced Planetary Science (CAPS), Guangzhou Institute of Geochemistry, Chinese Academy of Sciences, Guangzhou 510640, China; University of Chinese Academy of Sciences, Beijing 101408, China

**Keywords:** Chang'e-6, lunar samples, space weathering, space environment, lunar farside

## Abstract

The differences in terrain and chemical composition between the far side of the Moon (lunar farside) and the near side have been identified through remote sensing spectroscopy. The lunar farside samples returned by the Chang’e-6 mission show differences in terms of space weathering features compared to nearside samples. The studied farside samples lack vapor deposition layers found on the nearside and exhibit thinner amorphized layers, lower solar flare track densities, reduced number densities of nano phase metallic iron (npFe^0^) and larger npFe^0^ grain sizes. These findings suggest that the solar wind plays a dominant role in space weathering on the Chang'e-6 sampling site, surpassing micrometeorite impacts. This could provide critical sample-based evidence of the lunar space environment's dichotomy, enhancing our understanding of how solar wind and micrometeoroid impacts shape the lunar surface, even over short exposure periods.

## Teaser

The lunar farside samples returned by the Chang'e-6 mission show different space weathering features from that of the nearside samples, suggesting that the solar wind plays a dominant role in space weathering on the Chang'e-6 sampling site, surpassing micrometeorite impacts.

## INTRODUCTION

The Moon exhibits a distinct hemispherical dichotomy, with its nearside dominated by vast lunar mare and its farside characterized by rugged highlands and densely packed impact craters [1]. This dichotomy extends beyond surface morphology to differences in the Moon's space environment, a contrast well-established through remote sensing spectroscopic studies [2,3].

The unique spatial relationship within the Earth–Moon system results in significant environmental disparities between the nearside and farside. Since the Moon always keeps the same side facing Earth, the nearside periodically receives protection from Earth's magnetotail, partially shielding it from direct solar wind exposure. In contrast, the farside remains continuously exposed to solar wind, experiencing more intense radiation effects [4]. Additionally, meteoroid observations indicate that meteoroid radiants are not uniformly distributed, and the lunar surface is subject to sporadic meteoroids from fixed sources, such as the apex, helion, antihelion and northern toroidal sources, in addition to major meteoroid showers [2]. Consequently, due to orbital positioning and dynamic effects, the meteorite impact flux may also differ between the lunar nearside and farside [5].

These environmental differences profoundly affect the long-term evolutionary processes of the lunar surface. Due to the absence of an atmosphere and a global magnetic field, the lunar surface has been directly exposed to the harsh space environment, continuously subjected to micrometeoroid impacts and solar wind radiation. This suite of processes is collectively referred to as space weathering [6,7]. Space weathering serves as the dominant surface modification mechanism on airless bodies, with its features sensitively recording the environmental differences between the lunar nearside and farside [8,9]. Therefore, the unique microstructural features observed in lunar samples can reveal the differences in the lunar surface space environment. Studies of samples returned from the lunar nearside, as well as from the asteroids Itokawa and Ryugu, show that micrometeoroid impacts and solar wind radiation are the two primary processes driving space weathering. The formation of nanophase metallic iron (npFe^0^) due to these processes is the main factor responsible for significant changes in the Moon's optical reflectance spectra [9–12].

However, the landing sites of all 10 previous lunar sample-return missions, including Apollo, Luna and Chang'e-5, were confined to the lunar nearside. The sampling bias constrains our comprehension of the contrasting space weathering processes occurring between the lunar nearside and farside.

On 25 June 2024, the Chang'e-6 mission returned 1935.3 grams of lunar samples from the South Pole-Aitken Basin (41.625°S, 153.978°W) located on the farside of the Moon [13]. These lunar farside samples provide an opportunity for studying the effects of the space environment on space weathering.

This study focuses on the minerals in the scooped samples obtained from the Chang'e-6 mission to carry out a preliminary study on the space weathering features of various minerals. These minerals include silicate, sulfide and oxide minerals, all of which have undergone space weathering under the same space environment. By comparing the space weathering features of the lunar nearside and farside, we reveal the differences in space weathering processes of lunar samples exposed to distinct space environments and explore the relative contributions of solar wind and micrometeoroid impacts to the space weathering process. The results enhance our understanding of the dichotomy of the lunar space environment and provide valuable insights into how the lunar surface responds to the influence of different space environments.

## RESULTS

**Figure 1.**
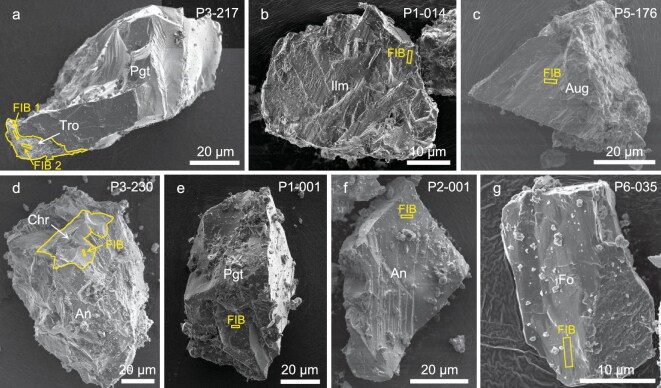
(a–g) Secondary electron (SE) images of the seven mineral clasts from the Chang'e-6 lunar farside sample investigated in this study. Yellow rectangles indicate the positions where FIB sampling was carried out and the curved yellow lines indicate mineral boundaries. Pgt, pigeonite; Tro, troilite; Ilm, ilmenite; Aug, augite; Chr, chromite; An, anorthite; Fo, forsterite.

Most particles in the Chang'e-6 samples exhibit surfaces coated with melt glass, which cements the particles together. However, melt drops and melt splashes resulting from micrometeorite impacts are relatively scarce (Fig. 1). We selected seven kinds of minerals (troilite, ilmenite, augite, chromite, pigeonite, anorthite and forsterite) with sharp, step-like edges, which were not covered by impact melts on their surfaces. Focused ion beam (FIB) technology was employed to extract eight sections (two sections from troilite: Fig. 1a–g; Table S1), then the microchemical and microstructural characteristics of space weathering of different kinds of minerals were systematically observed. All seven minerals show clear space weathering features under a scanning electron microscope (SEM) and transmission electron microscope (TEM), but they exhibit very diverse features and degrees of weathering.

**Figure 2.**
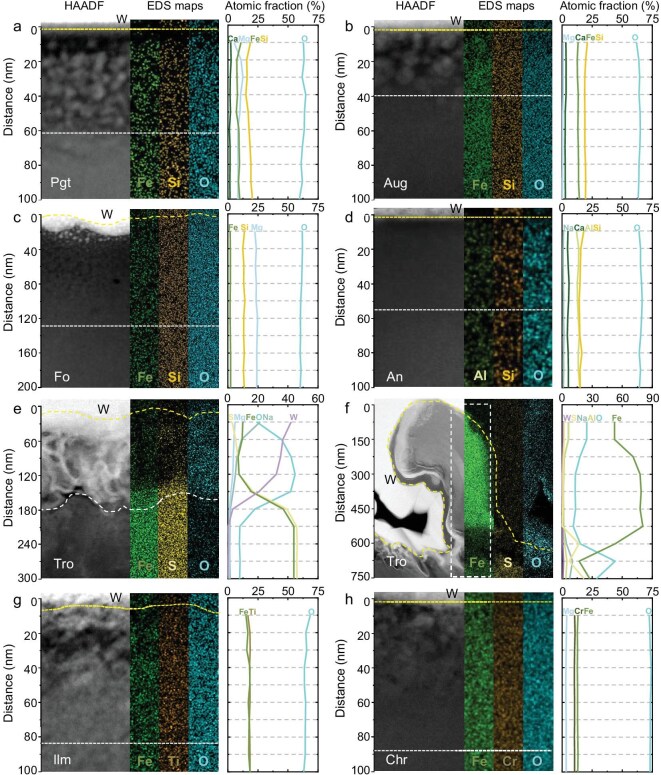
High-angle annular dark field (HAADF) images, energy dispersive spectroscopy (EDS) maps and corresponding EDS profiles of space weathering rims for eight focused ion beam (FIB) sections of the seven mineral particles from the Chang'e-6 lunar farside sample. (a–d) HAADF image and main elemental distribution maps of space weathering rims of pigeonite (Pgt), augite (Aug), forsterite (Fo), anorthite (An) and compositional variations of minerals near the surface. (e) HAADF image and main elemental distribution maps of the porous area of troilite (Tro), and compositional variations of minerals near the surface. (f) HAADF image and main elemental distribution maps of the iron whisker of troilite (Tro), showing compositional variations within the white dashed rectangle. (g–h) HAADF image and main elemental distribution maps of the space weathering rims of ilmenite (Ilm), chromite (Chr) and compositional variations of minerals near the surface. W, tungsten; C, carbon.

**Figure 3.**
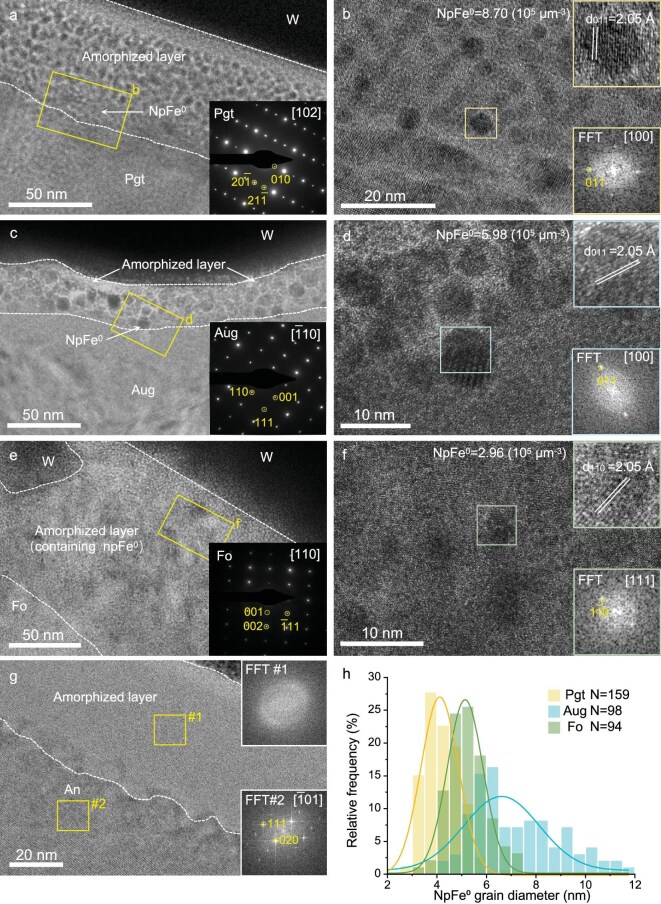
Microstructure and npFe^0^ grain statistics of space weathering rims of pigeonite, augite, forsterite and anorthite from the Chang'e-6 lunar farside sample. (a) High-resolution transmission electron microscope (HRTEM) image of the space weathering rims of pigeonite (Pgt) and its corresponding SAED pattern. (b) HRTEM image of npFe^0^ on the amorphized layer of pigeonite and its corresponding fast Fourier transform (FFT) pattern. (c) HRTEM image of the space weathering rims of augite (Aug) and its corresponding selected area electron diffraction (SAED) pattern. (d) HRTEM image of npFe^0^ on the amorphized layer of augite and its corresponding FFT pattern. (e) HRTEM image of the space weathering rims of forsterite (Fo) and its corresponding SAED pattern. (f) HRTEM image of npFe^0^ on the amorphized layer of forsterite and its corresponding FFT pattern. (g) HRTEM image of the space weathering rims of anorthite (An) and the corresponding FFT pattern of its amorphized layer and substrate. (h) Size and frequency histograms of npFe^0^ grains from space weathering in iron-bearing silicate minerals (pigeonite, augite and forsterite). W in the images represents the deposited tungsten.

For the silicate particles, energy dispersive spectroscopy (EDS) maps coupled with scanning transmission electron microscopy (STEM) show no significant compositional changes at the rims and interiors (Fig. 2a–d). In the high-angle annular dark field (HAADF)-STEM images of iron-bearing silicate minerals (pigeonite, augite and forsterite), many bright spots can be seen at the rims, with darker portions between them (Fig. 2a–c). These darker portions indicate that heavy elements (in this case iron) have been lost from the substrate minerals. However, there is no significant change in the corresponding EDS profiles (Fig. 2a–c), suggesting that iron in the substrate minerals underwent *in situ* segregation and aggregation within the layers, rather than migrating away from the surface (Figs S1–S3). High-resolution TEM (HRTEM) images show that the rims of all these silicate rims are amorphous, and that the round npFe^0^ disperse in a layer within the amorphized silicate layer. The npFe^0^ layer developed from the surface and its depth is less than the thickness of the amorphized layer. The thickness of the amorphized layer in pigeonite reaches 55 nm, and the depth of npFe^0^ is 50 nm; the amorphized layer in augite is 35 nm, and the depth of npFe^0^ is 30 nm; and the amorphized layer in forsterite is the widest, reaching 125 nm, but the depth of npFe^0^ is 50 nm (Fig. 3a, c, e). By measuring the lattice fringes of npFe^0^ in these silicates, it is recognized that all of these npFe^0^ are α-Fe (Fig. 3b, d, f).

In contrast, no npFe^0^ was observed in the iron-free anorthite, with only a 45 nm amorphized layer, which is thinner than those of pigeonite, augite and forsterite (Fig. 3g). The EDS data show that the amorphized zone of the anorthite has the same composition as the substrate crystalline zone, suggesting that the only difference between the rim and the substrate anorthite is that the rim lacks a long-range ordered crystal structure (Fig. 3g and Fig. S4).

**Figure 4.**
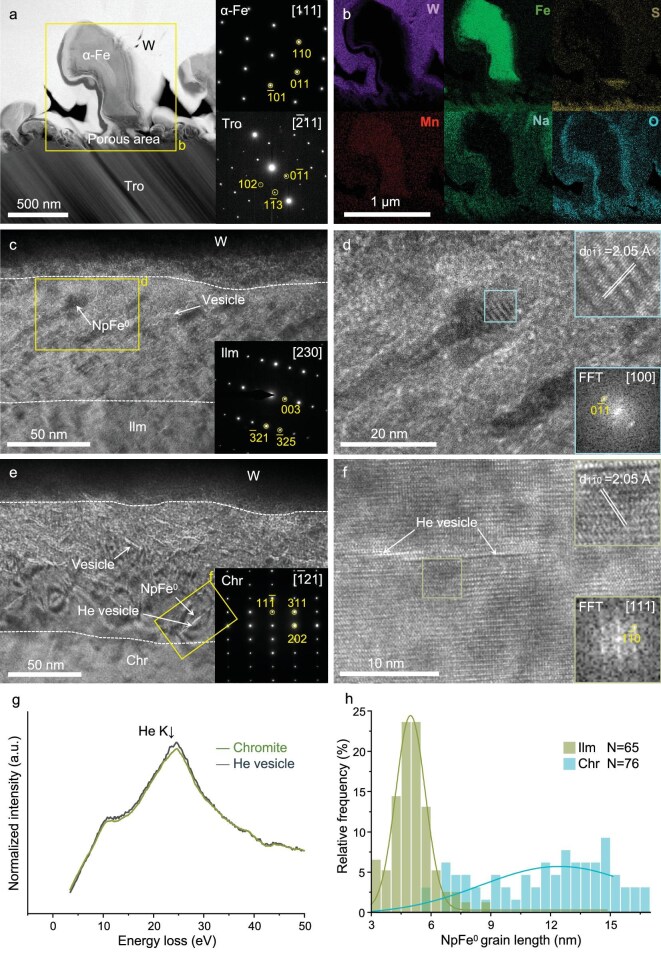
Microstructure and npFe^0^ grain statistics of the space weathering rims of troilite, ilmenite and chromite from the Chang'e-6 lunar farside sample. (a–b) HAADF image and EDS maps of troilite (Tro). Curtaining structures in the substrate troilite are artificial features produced during Ga^+^ ion milling. (c) HRTEM image of the space weathering rims of ilmenite and its corresponding SAED pattern. (d) HRTEM image of npFe^0^ of ilmenite (Ilm) and its corresponding FFT pattern. (e) HRTEM image of the space weathering rims of chromite (Chr) and its corresponding SAED pattern. (f) HRTEM image of npFe^0^ and the helium vesicle of chromite and its corresponding FFT pattern. (g) Selected EELS spectra from the helium vesicle region show the presence of a small amount of helium (22 eV) within some of the defects and from the chromite substrate. (h) Size and frequency histograms of npFe^0^ grains from the space weathering rims in Chang'e-6 ilmenite and chromite. W in the images represents the deposited tungsten.

For troilite, SEM analysis reveals a porous structure consisting of aggregates of needle-like material on the troilite surface, with some regions containing curled iron whiskers of varying sizes. We extracted samples from the porous and iron whisker areas using FIB (Fig. 1a and Fig. S5a and b). The thickness of the porous area is ∼150 nm, and the rim of the FIB section is brighter under the HAADF image and can be clearly distinguished from the substrate troilite due to a certain amount of tungsten deposited in the pores during the sampling process (Fig. 2e). EDS analysis showed that oxygen, sodium and magnesium were enriched in the porous area (Fig. S5). In addition, there are simultaneous losses of iron and sulfur in the porous area, but the Fe/S ratio in the porous area is higher than that in the substrate area, indicating that the loss of sulfur is greater than the loss of iron (Fig. 2e and f). Sputtering due to solar wind radiation, and chemical reactions of the sample with hydrogen ions from the solar wind may lead to selective loss of sulfur from sulfides, resulting in the formation of porous regions on the surface of the troilite and the high Fe/S ratios detected in this region [14]. The selective loss of sulfur leads to the accumulation of excess Fe^2+^ in the damaged rims of the troilite. Given that the solar wind contains electrons and troilite possesses excellent electrical conductivity, the excess Fe^2+^ may be reduced to Fe^0^ by free electrons near the surface (Fe^2+^ + 2e^−^ = Fe^0^). Continued loss of sulfur and reduction of Fe^2+^ may lead to the growth of metal nuclei on the sulfide surface and the formation of iron whiskers from the substrate troilite [15,16]. The largest iron whisker in the FIB section is ∼1 μm high and ∼300 nm in diameter (Fig. S5c and d), and selected area electron diffraction (SAED) reveals the iron whisker as α-Fe (Fig. 4a). EDS mapping shows that the root of the iron whisker (at the junction with troilite) exhibits elemental distributions consistent with the porous area, where it is enriched in oxygen and sodium, and has a higher Fe/S ratio than that of the substrate troilite (Figs 2f and 4b). The iron whiskers have ∼50–100 nm oxygen-enriched surfaces, and trace amounts of manganese are uniformly distributed within the iron whiskers (Fig. 4b).

For iron oxide minerals (ilmenite and chromite), irregular rims were observed in their FIB sections. Within these rims, vesicles were identified that are absent in silicate minerals (Fig. 4c and e). These vesicles distributed to a depth of ∼100 nm below the surface of the grain rims and generally ranged in size from 5 to 20 nm. These vesicles caused the sample surface to swell, forming a ‘fine spotted structure’ (blisters) under SEM observation (Fig. S6a and b). The EDS data indicate that the rims, interiors and vesicle regions have the same composition, suggesting that the only difference between the vesicles and the other sites is that their crystal lattice was distorted (Fig. 4c–f; Figs S7 and S8). In chromite, helium vesicles up to 20 nm long were also found, and the peaks of helium were visible in the electron energy loss spectroscopic (EELS) spectrum (Fig. 4g), suggesting that they were formed by lattice damage caused by helium injection [17]. Additionally, irregular npFe^0^ particles can be observed near the vesicles, and the shape of the npFe^0^ particles is sometimes controlled by the crystal structure of ilmenite and chromite, which indicates the crystal orientation (Fig. 4d and f).

## DISCUSSION

### Heterogeneity in the degree of space weathering of the Moon

Based on the results from the Chang’e-6 samples, several key differences in space weathering characteristics are clearly evident when compared to other lunar samples such as those from Chang’e-5 and Apollo missions, as well as asteroid samples from Itokawa [9,18–20].

**Figure 5.**
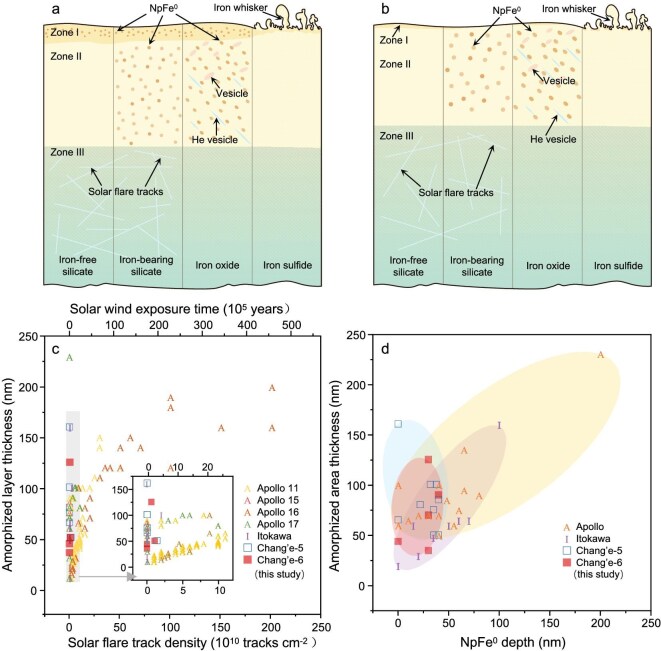
Comparative plots of space weathering rim features in this study versus lunar nearside and Itokawa samples. (a) Schematic of space weathering features in essential minerals from the lunar nearside and Itokawa samples. (b) Schematic of space weathering features in essential minerals from the samples in this study. (c) Scatter plot of solar-flare track density versus amorphized layer thickness in lunar and Itokawa samples. The upper axis shows the solar wind exposure time calculated based on the solar-flare track production rate on the lunar surface (1 AU is 4.4 ± 0.4 × 10^4^ tracks cm^−2^ year^−1^, [26]). (d) Scatter plot of npFe^0^ depth versus amorphized layer thickness in lunar and Itokawa samples. Zone I, vapor deposition zone; Zone II, solar wind damage zone; Zone III, crystalline substrate zone. Chang'e-5 data from [18,41], Apollo data from [17,26,42–45] and Itokawa data from [20,25,46,47].

In the Chang'e-6 sample, we did not observe a significant vapor deposition layer, or enrichment of oxygen, sodium and magnesium elements that were not from substrate minerals, which was observed only in porous areas on the surface of troilite (Fig. 2e; Fig. S5). The distribution of oxygen detected in the porous area of troilite may be due to oxidation on the lunar surface or during sample processing on Earth [21]. Other elements may represent vapor deposits from micrometeorite impacts [22]. All other space weathering features, including amorphized layers, vesicles and npFe^0^, with compositions consistent with the substrate mineral compositions, can be attributed to damage due to solar wind radiation. In contrast, lunar nearside samples from the Chang’e-5 and Apollo missions exhibit both a vapor deposition zone (Zone I) and a solar wind damage zone (Zone II) (Fig. 5a and b).

The Chang'e-6 samples exhibit a thinner amorphized layer, a feature directly associated with their shorter exposure to solar wind. The Chang’e-6 samples have a younger rock age compared to Apollo samples [23,24], and the lower density of solar flare tracks in the Chang’e-6 samples provides further evidence of their shorter solar wind exposure time. We estimated the solar wind exposure time of the samples in this study from solar wind damage on the surfaces of silicate minerals. The solar flare tracks beneath the amorphized layers of forsterite and pigeonite. The density of solar-flare tracks under the amorphized layer of forsterite is statistically 0.62 × 10^10^ tracks cm^−2^ and under that of pigeonite is 1.08 × 10^10^ tracks cm^−2^ (Fig. S9). The density of solar-flare tracks is close to the minimum observed in the Apollo 11 sample, smaller than the values in other Apollo samples, and slightly smaller than the Chang'e-5 sample (1.4 × 10^10^ tracks cm^−2^ [18]) and Itokawa sample (2.0 × 10^10^ tracks cm^−2^ [25]). Solar wind exposure times, calculated using solar-flare track densities in forsterite and pigeonite, are 1.4 × 10^5^ and 2.4 × 10^5^ years, respectively (the track production rate at 1 AU is 4.4 ± 0.4 × 10^4^ tracks cm^−2^ year^−1^ [26]). The shorter solar wind exposure time may account for the smaller range of amorphized layer thicknesses in these samples relative to the Chang'e-5 and Apollo samples (Fig. 5c), as well as affecting the accumulation of npFe^0^ and vesicle at the space weathered rims (Fig. 5d).

Amorphization at the rim of silicate minerals gives the *in situ* isolated npFe^0^ a spherical shape, while ilmenite and chromite, which are more resistant to radiation-induced amorphization, result in preferentially oriented and elongated npFe^0^ in the solar wind damage zone. NpFe^0^ is more abundant in low-Ca, high-Fe pigeonite, with an average grain size of 4.2 ± 0.7 nm (Fig. 3h) and a number density of 8.70 × 10^5^ μm^−3^ (Fig. 3b). In contrast, npFe^0^ grains are larger in high-Ca, low-Fe augite, exhibiting an average grain size of 7.2 ± 1.8 nm (Fig. 3h) and a density of 5.98 × 10^5^ μm^−3^ (Fig. 3d). Additionally, npFe^0^ in low-Fe forsterite has an average grain size of 5.2 ± 0.8 nm (Fig. 3h). But its depth is limited to 35 nm, and the density is 2.96 × 10^5^ μm^−3^ (Fig. 3f). The npFe^0^ observed in this study are coarser than previous reports including the 2.6 nm average size of npFe^0^ in the Apollo samples, the 5 nm average size in the Chang'e-5 samples, and the 2 nm average size in the Itokawa sample (Table S2) [18,20]. The length of the npFe^0^ particles in chromite has a single-peaked normal distribution, while the lengths of npFe^0^ in ilmenite are bimodal and exhibit lower number densities (Fig. 4h). The npFe^0^ grains are believed to have formed through solar wind radiation damage rather than vapor deposition, as they are found in rims with the same composition as the substrate minerals, without the incorporation of foreign depositional material [27].

### Space-environment-driven differences in space weathering

The aforementioned discussion accounts for a distinctive difference in space weathering features between Chang'e-6 and previous lunar and asteroid samples. The differences in space weathering characteristics between the lunar farside and nearside samples can be attributed to several factors including lunar magnetic anomalies, physical properties of lunar regolith and space environments. Although magnetic anomalies on the lunar surface could potentially enhance or suppress space weathering processes [28,29], we will not discuss this topic as the Chang'e-6 sampling site is not located in a zone with significant magnetic anomalies [30,31].

The physical properties of the Chang'e-6 samples differ from those of other lunar samples. The lower bulk density of the Chang'e-6 sample (0.75 g cm^−3^) [13], which is close to the minimum of the Apollo 11 sample, is much smaller than that of the other Apollo and Chang'e-5 samples (1.1–2.1 g cm^−3^) [32,33]. This suggests that the Chang'e-6 lunar soil is looser and more porous. Impact on a more porous sample generates more heat as the shock energy is expended in collapsing the pores [34]. Therefore, the Chang'e-6 sample would have experienced higher post-shock temperatures after impact with impactors of the same velocity and volume, which could allow the particles to acquire higher thermal energy, and become further enlarged by heating after the initial formation of the npFe^0^ grains. Impact heating simulations by Thompson *et al.* [35] demonstrate that the original npFe^0^ grain size within minerals increases with elevated temperatures. The interlaced, spotty and splashed melt observed on the particle surfaces suggests a complex impact history for the Chang'e-6 samples (Fig. S10a and b). Furthermore, the impact history may have contributed to the observed variations in space weathering characteristics [36]. The impact events likely produced fresh surfaces with shorter solar wind exposure durations, leading to the thinner amorphized layers identified in this study.

**Figure 6.**
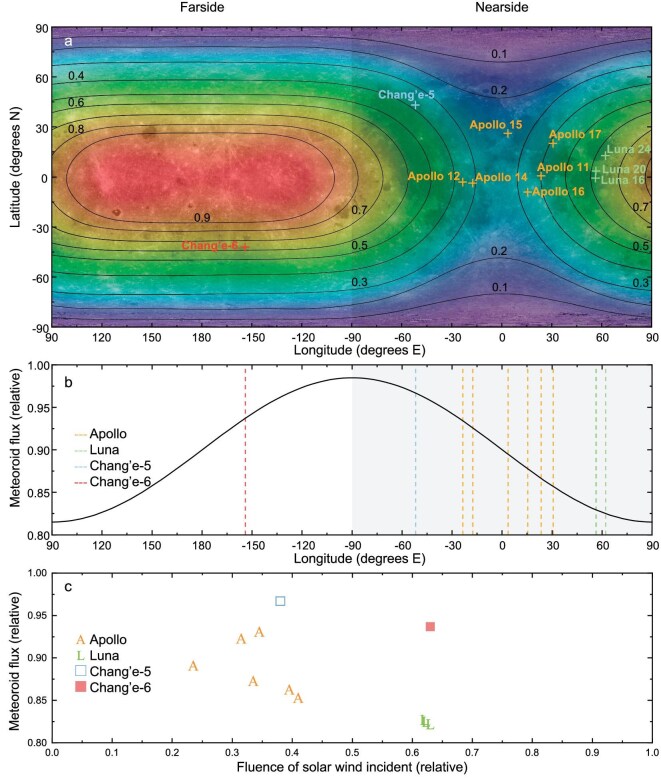
Relative fluence of solar wind action and (micro)meteorite impacts at different locations on the Moon. (a) The relative fluence of solar wind on different lunar locations, based on modifications from [8,37]. (b) The relative (micro)meteoroid flux at different longitudes was calculated using data from the Lunar Atmosphere and Dust Environment Explorer's Lunar Dust Experiment (LADEE LDEX) by [5] and modified from [8]. (c) The relative contribution of solar wind and (micro)meteoroid impact at different lunar sampling sites.

A more important point is that different latitudinal and longitudinal locations on the lunar surface are exposed to varying solar wind influences and meteoroid impact fluxes. The Earth's magnetotail significantly affects the solar wind flux reaching the lunar surface, with the Moon entering the magnetotail during each synodic month. This results in a reduction when, after time, the lunar surface is exposed to the solar wind [4,37]. In contrast, the lunar farside does not enter the magnetotail region during each synodic month cycle and is therefore not shielded by the Earth's magnetic field. As a result, the far side of the Moon is exposed to stronger solar wind radiation (Fig. 6a). Therefore, minerals in the farside samples exhibit distinct solar-wind-induced damage layers on their surfaces. The damage layers and vesicle depths in oxides are greater than those observed in Apollo and Chang'e-5 samples [17,18]. Additionally, the larger npFe^0^ grain size and lower density observed in the Chang'e-6 samples suggest more pronounced iron segregation and aggregation caused by solar wind radiation. This is supported by studies on lunar swirls [9] and poleward-facing crater walls [38], which exhibit higher albedo than their surroundings in areas where solar-wind flux is reduced, though micrometeoroid flux and the local environment remain unchanged. These findings suggest that while micrometeorite impacts can produce some metallic iron, the greater abundance or size of npFe^0^ requires interaction with the solar wind. Solar wind simulation experiments by Loeffler *et al.* [39] and Christoffersen *et al.* [40] have demonstrated that npFe° can form during ion irradiation, underscoring the critical role of the solar wind in lunar space weathering. These results indicate that the solar wind significantly influences the space weathering features observed in Chang'e-6 samples.

The lunar surface is also subjected to continuous impacts from sporadic meteoroids, which originate from comets and asteroids. The relative velocity between the Moon and the meteoroids varies with the lunar phases: during a full moon, the Moon and the meteoroids move in the same orbital direction, increasing their relative velocity. During a new moon, the Moon's orbital motion is opposite to that of the meteoroids, reducing their relative velocity [5]. Therefore, the meteoroid flux is influenced by the Moon's orbital characteristics, leading to variations in flux across different regions (Fig. 6b). Sharp surface steps are observed on minerals in the Chang'e-6 samples, indicating surface fragmentation and cleavage fractures caused by intense micrometeorite impacts. However, no vapor deposition layers formed by high-temperature condensates from micrometeorite impacts were detected. Unlike Chang'e-5 samples, no impact-generated npFe^0^ particles were found in the space weathered rims of the Chang'e-6 samples [41].

Solar wind radiation and micrometeorite impacts are the two most dominant drivers of space weathering, but data from Keller and McKay [22] suggest that the effective sputtering rate from the solar wind and vapor deposit from micrometeorite impacts offset each other. When discussing the causes of space weathering, it is important to consider the relative contributions from solar wind radiation and micrometeoroid impacts in different space environments (Fig. 6c). The space weathering features observed in Chang'e-6 samples indicate that the contribution of the solar wind surpasses that of micrometeoroid impacts in lunar farside samples. In contrast, reports on Chang'e-5 and Apollo samples suggest that micrometeoroid impacts play a more dominant role than the solar wind. This provides a key indicator of space weathering differences driven by the lunar space environment.

## CONCLUDING REMARKS

In summary, we performed a preliminary analysis of space weathering features of lunar farside samples returned by the Chang'e-6 mission. Our results emphasize the dominant role of solar wind radiation in space weathering processes on the lunar farside. By comparing the Chang'e-5, Apollo samples from the lunar nearside, and samples from the airless asteroid Itokawa, we observed that the studied Chang'e-6 lunar farside samples lack distinct vapor deposition layers, have relatively thinner amorphized layers, exhibit lower densities and larger grain sizes of npFe^0^ in iron-bearing minerals. These differences could highlight the significant role of space environmental variables, offering valuable insights into the variations in space weathering processes influenced by the lunar space environment.

Nonetheless, we must bear in mind that the randomness in sample selection may bring about some uncertainty in the study. The inherent heterogeneity of lunar regolith means that individual samples may not fully represent the broader features of the lunar surface. Although we have comprehensively examined the essential minerals to provide a complete understanding of how different mineral types are affected by space weathering, the observed differences may still be influenced by the studied specific samples. To overcome this constraint, future research should expand the number of samples and encompass a broader range of geographical locations to ensure the findings are comprehensive and reliable.

## MATERIALS AND METHODS

### Samples

The lunar soil sample (CE6C0400YJFM003) used in this study was returned by the Chang'e-6 mission and provided by the China National Space Administration (CNSA). Powder samples were processed in a glove box (N_2_ > 99.999%, H_2_O < 0.01 ppm, O_2_ < 1.0 ppm, Mikrouna). Selected powder samples were dispersed on a conductive adhesive and coated with 10 nm carbon, by using a Leica EM ACE600 coater, for further surface morphological observation by SEM.

### Scanning electron microscope observations

A conductive adhesive with powder lunar samples was transferred in the air to a JEOL JSM-IT-800 SEM for observation. The secondary electron images were collected at low voltage (3 kV) with 0.34 nA emission current. Observation under a low voltage made electron irradiation damage to the sample surfaces as low as possible. During the SEM observations, potential positions for FIB cutting were recorded (Fig. 1).

### FIB sampling and transmission electron microscopy analyses

After the SEM observations, the sample was transferred to a Thermo Fisher Scientific FEI Helios CX5 dual-beam system. The recorded positions for FIB cutting were deposited with tungsten. Thin sections for TEM observations were then sampled by a 30 kV Ga^+^ ion beam in the FIB system. The FIB thin section was then loaded into a Thermo Fisher Scientific FEI Talos F200S TEM (operated at 200 kV) and observed in TEM and STEM modes at the Electron Microscopy Center of Guangzhou Institute of Geoch emistry, Chinese Academy of Sciences (CAS).

In the TEM mode, bright field images and SAED patterns were acquired using a Ceta-16M camera. In the STEM mode, images were acquired using a HAADF detector. EDS mapping was performed in STEM mode with two superX detectors. The dwell time was 2 μs, and the results were summed from 150–500 frames. EDS semi-quantitative analyses from mapping data were conducted using the FEI Velox software. EELS analyses were performed in STEM mode with a Gatan 1077 EELS spectrometer. The pixel step for acquiring a spectroscopic image was 1 nm. All the data were acquired in a dual EELS mode with zero-peak locking. All the EELS data processing tasks (including background subduction, signal integration, data fitting and mapping) were conducted in the Gatan Microscope Suite (GMS) software (version 3.50).

## Supplementary Material

nwaf087_Supplemental_File

## Data Availability

The experiment data that support the findings of this study are available via the Science Data Bank repository at https://www.scidb.cn/en/anonymous/dUlicU1q.
