# Supplementary material for: Differences in space weathering between the near and far side of the Moon: evidence from Chang'e-6 samples
Source: Natl Sci Rev. 2025 Mar 5;12(6):nwaf087. doi: 10.1093/nsr/nwaf087 (PMC12051863; doi:10.1093/nsr/nwaf087)
Supplement: nwaf087_Supplemental_File [file nwaf087_supplemental_file.docx]

**Supplementary Data for**

**Differences in space weathering between the near and far side of the Moon: Evidence from Chang'e-6 samples**

Jiarui Lin^1,2,3,4^, Haiyang Xian^1,2,3^*, Yiping Yang^1,2,3^, Shan Li^1,2,3,4^, Jiaxin Xi^1,2,3,4^, Xiaoju Lin^1,2,3^, Yao Xiao^1,2,3,4^, Shengdong Chen^1,2,3,4^, Chenyi Zhao^1,2,3^, Miaomiao Zhang^1,2,3^, Akira Tsuchiyama^1,2^, Jianxi Zhu^1,2,3^*, Hongping He^1,2,3,4^, and Yi-Gang Xu^1,2,4^

^1^ State Key Laboratory of Deep Earth Processes and Resources, Guangzhou Institute of Geochemistry, Chinese Academy of Sciences, Guangzhou, 510640, China.

^2^ Center for Advanced Planetary Science (CAPS), Guangzhou Institute of Geochemistry, Chinese Academy of Sciences, Guangzhou, 510640, China.

^3^ Guangdong Provincial Key Laboratory of Mineral Physics and Materials, Guangzhou Institute of Geochemistry, Chinese Academy of Sciences, Guangzhou, 510640, China.

^4^ University of Chinese Academy of Sciences, Beijing, 101408, China.

^∗^Corresponding authors. E-mails: [xianhaiyang@gig.ac.cn](mailto:xianhaiyang@gig.ac.cn) (Haiyang Xian), [zhujx@gig.ac.cn](mailto:zhujx@gig.ac.cn) (Jianxi Zhu)

**This file includes:**

Figures S1: HAADF image and EDS maps of the pigeonite FIB section.

Figures S2: HAADF image and EDS maps of the augite FIB section.

Figures S3: HAADF image and EDS maps of the forsterite FIB section.

Figures S4: HAADF image and EDS maps of the anorthite FIB section.

Figures S5: SE image, HAADF image and EDS maps of the troilite FIB section.

Figures S6: SE image of the surface of ilmenite and chromite

Figures S7: HAADF image and EDS maps of the ilmenite FIB section.

Figures S8: HAADF image and EDS maps of the chromite FIB section.

Figures S9: Bright-field TEM image of the pigeonite and forsterite.

Figures S9: Bright-field TEM image of the pigeonite and forsterite.

Figures S10: SE image of splashed melt on pigeonite and anorthite surfaces.

Table S1. The EDS chemical compositions of various mineral phases.

Table S2. npFe^0^ grain size for space weathering layers of each mineral in different samples.

Supplementary References.


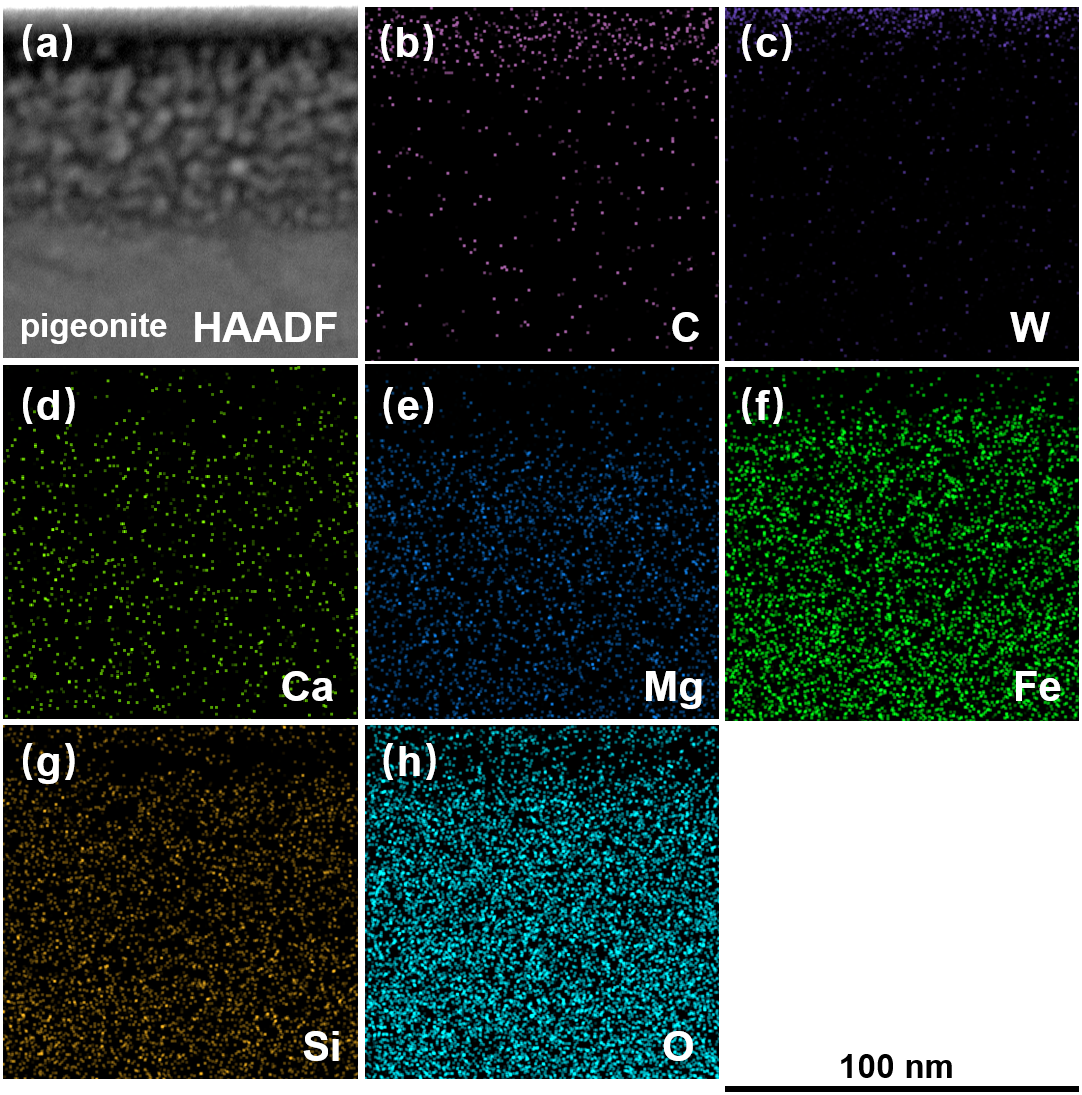


Figure S1. (a-h) HAADF image and EDS maps of the pigeonite FIB section.


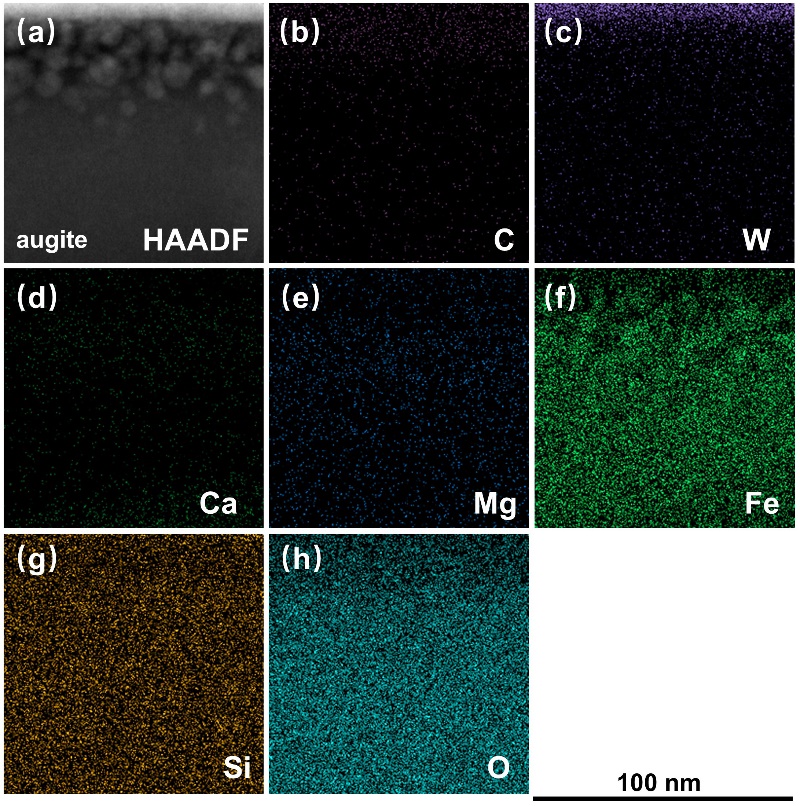


Figure S2. (a-h) HAADF image and EDS maps of the augite FIB section.


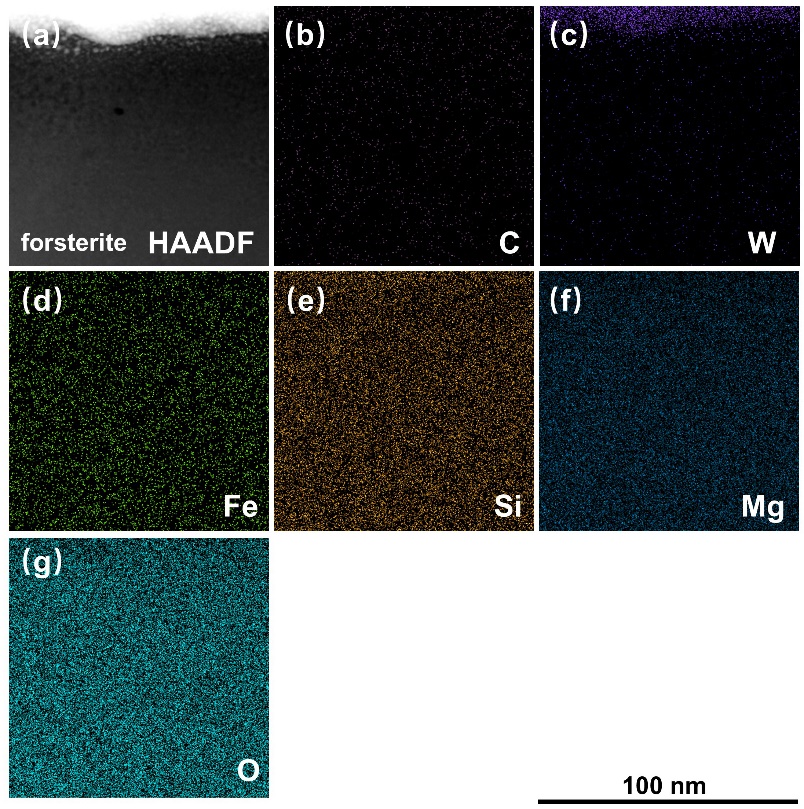


Figure S3. (a-g) HAADF image and EDS maps of the forsterite FIB section.


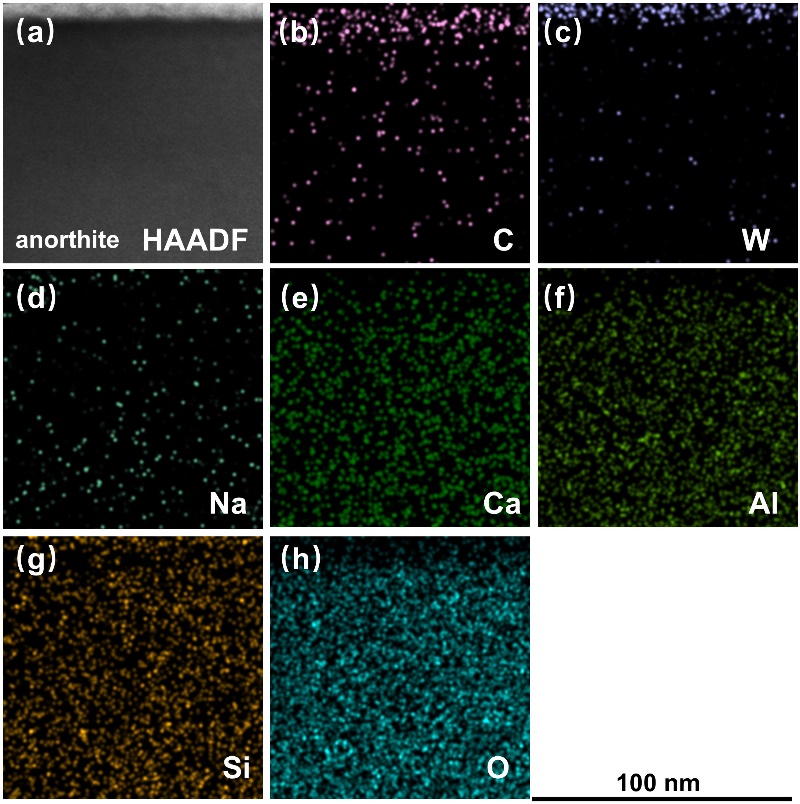


Figure S4. (a-h) HAADF image and EDS maps of the anorthite FIB section.


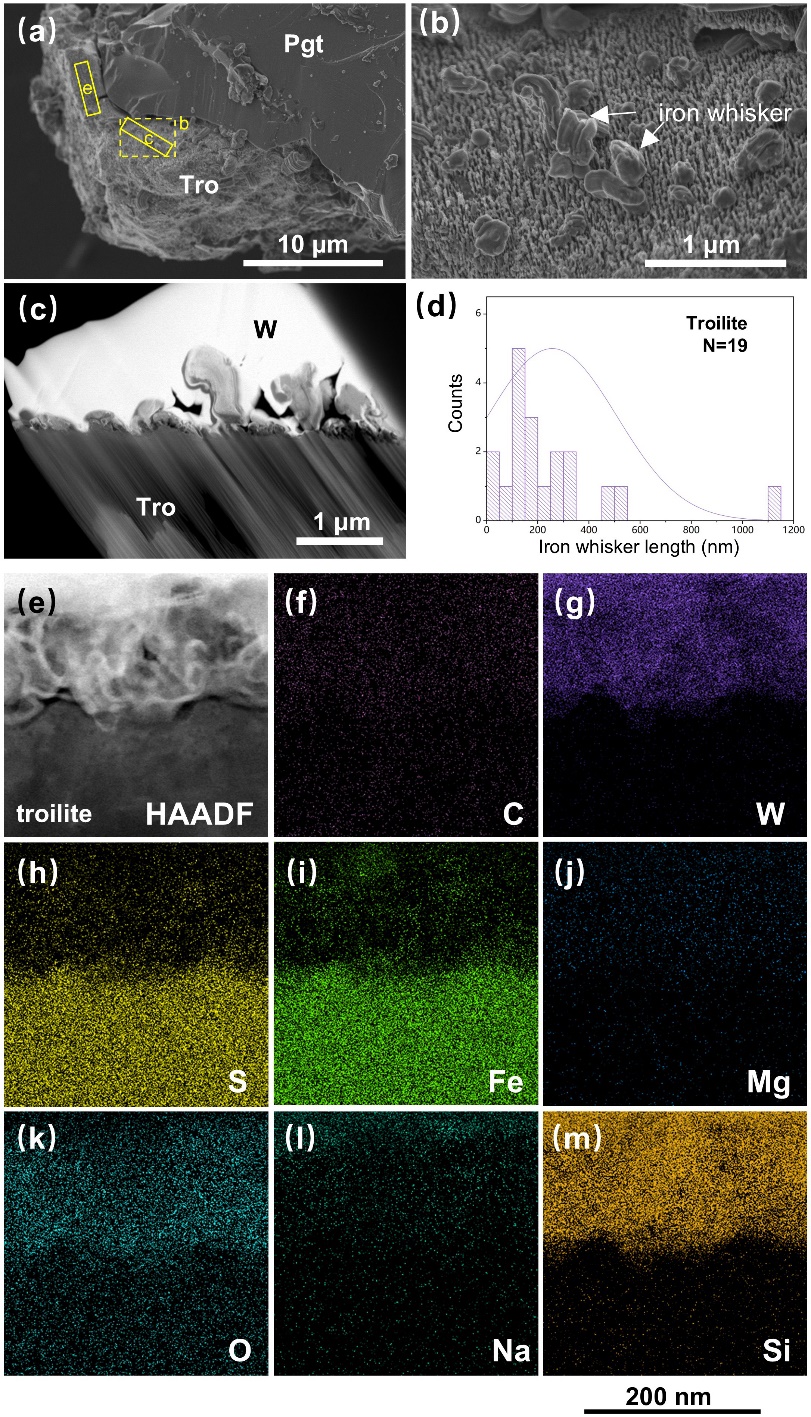


Figure S5. (a) SE image of the surface of troilite (Tro). (b) is a magnified image of the dashed rectangle in (a). (c) is the HAADF image of the FIB section extracted from the solid rectangle c in (a). (d) Size histograms of iron whisker from space weathering in troilite. (e) is the HAADF image of the FIB section extracted from the solid rectangle e in (a). (f-m) EDS maps of the troilite FIB section.

Pgt, pigeonite; W, tungsten.


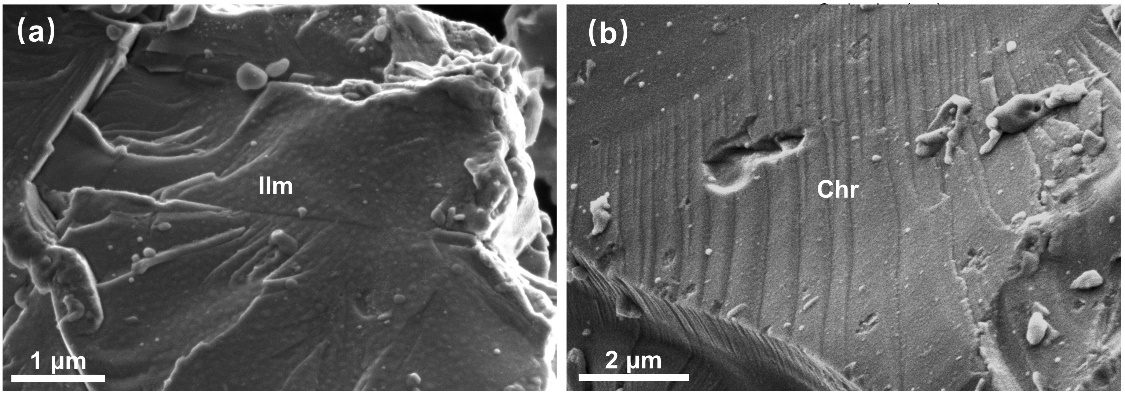


Figure S6. (a) SE image of the surface of ilmenite (Ilm). (b) SE image of the surface of chromite (Chr).


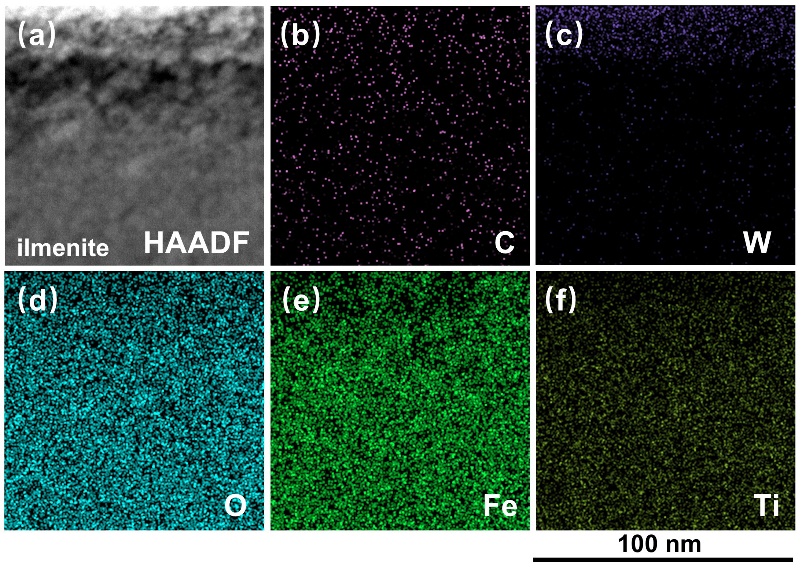


Figure S7. (a-f) HAADF image and EDS maps of the ilmenite FIB section.


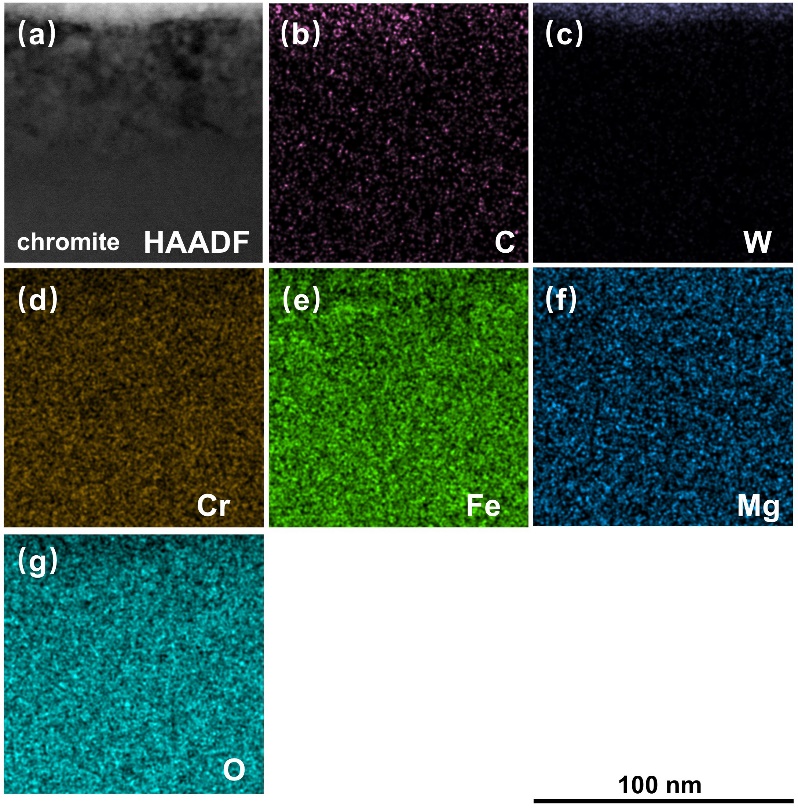


Figure S8. (a-g) HAADF image and EDS maps of the chromite FIB section.


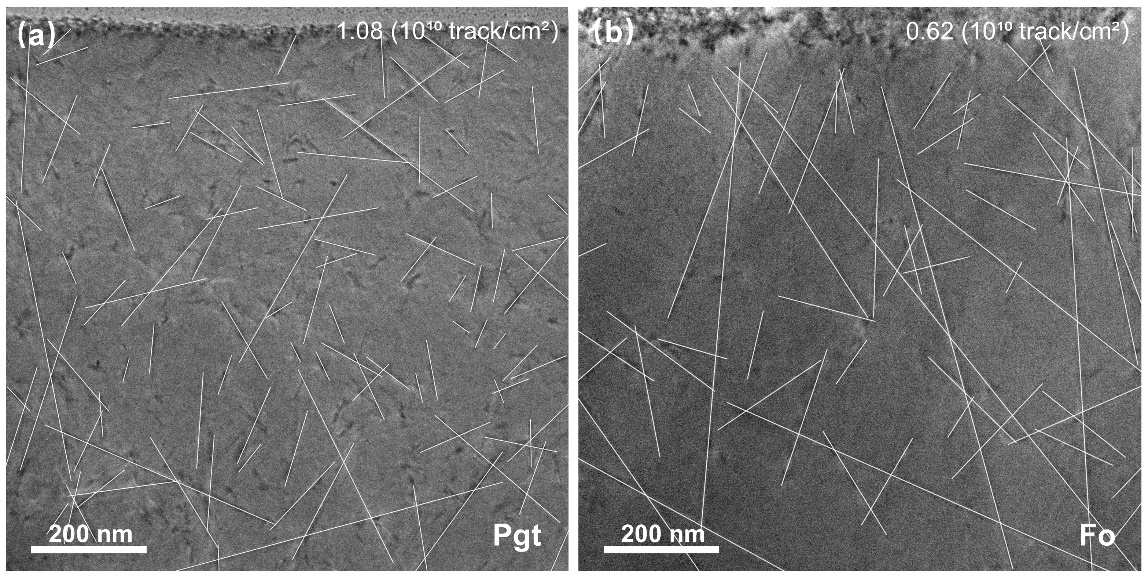


Figure S9. Bright-field TEM image of the pigeonite (Pgt) and forsterite (Fo), showing the upper amorphized area and the solar flare tracks.


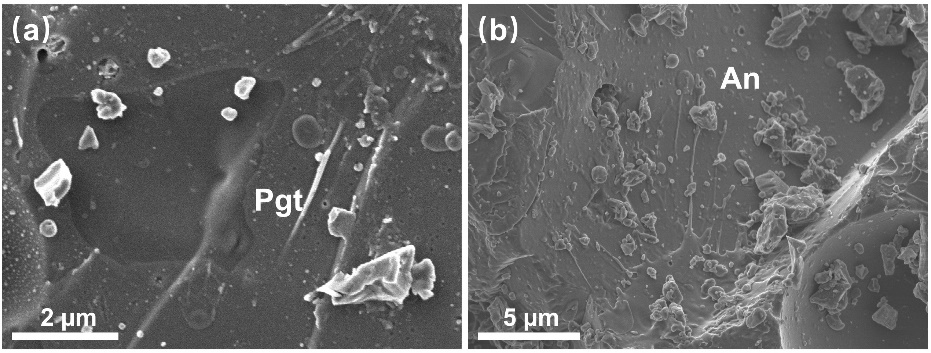


Figure S10. SE image of splashed melt on pigeonite (Pgt) and anorthite (An) surfaces.

Table S1. The EDS chemical compositions (in at%, normalized to 100%) of various mineral phases.

| No. | Sample | O | Na | Mg | Al | Si | Ca | Fe | S | Ti | Cr |
| --- | --- | --- | --- | --- | --- | --- | --- | --- | --- | --- | --- |
| P1-001 | Pgt | 58.87 | bdl^a^ | 9.17 | 1.06 | 20.39 | 1.62 | 8.89 | bdl | bdl | bdl |
| P1-014 | Ilm | 66.1 | bdl | bdl | 0.44 | 0.73 | 16.81 | 15.92 | bdl | bdl | bdl |
| P2-001 | An | 65.53 | 1.19 | 9.57 | 14.05 | 15.65 | 4.84 | 0.18 | bdl | bdl | bdl |
| P3-217 | Tro-1 | bdl | bdl | bdl | bdl | bdl | bdl | 50.1 | 49.9 | bdl | bdl |
| P3-217 | Tro-2 | bdl | bdl | bdl | bdl | bdl | bdl | 48.63 | 51.37 | bdl | bdl |
| P3-230- | Chr | bdl | bdl | bdl | bdl | bdl | bdl | 12.6 | 12.64 | 4.72 | 10.8 |
| P5-176 | Aug | 65.77 | bdl | 1.54 | 0.85 | 18.14 | 6.26 | 7.45 | bdl | bdl | bdl |
| P6-035 | Fo | 60.69 | bdl | 23.98 | bdl | 13.25 | bdl | 2.08 | bdl | bdl | bdl |

^a^ Note: “bdl” indicates that no element was detected.

Pgt, pigeonite; Tro, troilite; Ilm, ilmenite; Aug, augite; Chr, chromite; An anorthite; Fo, forsterite.

Table S2. npFe^0^ grain size (nm) for space weathering layers of each mineral in different samples.

| Sample | Hpx | Lpx | Ol | Ilm | Chr | References |
| --- | --- | --- | --- | --- | --- | --- |
| Apollo 11 | -^a^ | - | - | 14.8 | - | [1] |
| Apollo 15 | 2.82 | - | - | - | - | [2] |
| Apollo16 | - | - | 2.27 | 7.88 | - | [1,3] |
| Apollo 17 | 3.33 | - | - | 7.9 | 2.73 | [4] |
| Itokawa | 2.2 | 2.4 | 1.5 | - | - | [5] |
| Chang'e 5 | 3.5 | 4.6 | 6.1 | 11.7 | - | [6,7] |
| Chang'e 6 | 4.2 | 7.2 | 5.2 | 11.8 | 5.1 | This study |

^a^Note: “-” indicates that no relevant data were collected.

Hpx, high-Ca pyroxene; Lpx, low-Ca pyroxene; Tro, troilite; Ilm, ilmenite; Chr, chromite.

**Supplementary References**

1. Christoffersen R, McKay DS, Keller LP. Microstructure, chemistry, and origin of grain rims on ilmenite from the lunar soil finest fraction. *Meteorit & Planetary Scien* 1996;31:835-848.

2. Gu L, Zhang B, Hu S, et al. The discovery of silicon oxide nanoparticles in space-weathered of Apollo 15 lunar soil grains. *Icarus* 2018;303:47-52.

3. Keller LP, Berger EL, Zhang S, et al. Solar energetic particle tracks in lunar samples: A transmission electron microscope calibration and implications for lunar space weathering. *Meteorit & Planetary Scien* 2021;56:1685-1707.

4. Burgess KD, Stroud RM. Phase-dependent space weathering effects and spectroscopic identification of retained helium in a lunar soil grain. *Geochimica et Cosmochimica Acta* 2018;224:64-79.

5. Noguchi T, Kimura M, Hashimoto T, et al. Space weathered rims found on the surfaces of the Itokawa dust particles. *Meteorit & Planetary Scien* 2014;49:188-214.

6. Gu L, Chen Y, Xu Y, et al. Space Weathering of the Chang’e‐5 Lunar Sample From a Mid‐High Latitude Region on the Moon. *Geophysical Research Letters* 2022;49:e2022GL097875.

7. Guo Z, Li C, Li Y, et al. Nanophase Iron Particles Derived From Fayalitic Olivine Decomposition in Chang’E‐5 Lunar Soil: Implications for Thermal Effects During Impacts. *Geophysical Research Letters* 2022;49:e2021GL097323.
